# Combined Assessment of Immunonutritional Indices and the Triglyceride-Glucose Index in Coronary Slow Flow Phenomenon in a Non-Elderly Population

**DOI:** 10.3390/jcm15114004

**Published:** 2026-05-22

**Authors:** Cagdas Kaynak, Muzaffer Aslan

**Affiliations:** Department of Cardiology, Siirt Education and Research Hospital, Siirt 56100, Turkey; mzfraslan56@hotmail.com

**Keywords:** insulin resistance, Prognostic Nutritional Index, nutritional risk, triglyceride-glucose index, coronary microvascular dysfunction, risk stratification, coronary slow flow phenomenon

## Abstract

**Background/Objectives:** Coronary slow flow phenomenon (CSFP) is considered a condition identified during coronary angiography (CAG), associated with recurrent ischemic symptoms and adverse cardiovascular outcomes while no significant epicardial coronary obstruction is present. The combined predictive role of metabolic and nutritional indices in CSFP has not been fully elucidated. **Methods:** This analysis, based on a retrospective observational design at a single center, included 214 patients aged < 65 years undergoing CAG who had either normal coronary arteries (NCA) (*n* = 100) or CSFP (*n* = 114). CSFP was defined using the TIMI frame count criteria. The triglyceride-glucose (TyG) index, Prognostic Nutritional Index (PNI), Controlling Nutritional Status (CONUT) score, Naples prognostic score (NPS), and C-reactive protein–albumin–lymphocyte (CALLY) index were calculated. Logistic regression was employed to assess independent contributors to CSFP. **Results:** In comparison with the NCA group, patients with CSFP were more frequently male (73.7% vs. 43.0%, *p* < 0.001) and active smokers (33.3% vs. 19.0%, *p* = 0.018). Among these calculated indices, higher TyG index values were observed; in contrast, the CSFP group exhibited lower PNI and CALLY index values. In multivariable analysis, male sex (OR = 5.187, 95% CI: 2.520–10.674, *p* < 0.001), the TyG index (OR = 1.811, 95% confidence interval [CI]: 1.251–2.622, *p* = 0.002), and PNI (OR = 0.544, 95% CI: 0.362–0.817, *p* = 0.003) retained their predictive value for CSFP. **Conclusions:** Coronary slow flow phenomenon in a non-elderly cohort appears to be linked to metabolic dysfunction, immunonutritional imbalance, and sex-specific differences, with the combined evaluation of the TyG index, PNI, and male sex potentially enhancing risk stratification.

## 1. Introduction

When obstructive epicardial coronary pathology is not present, delayed opacification of distal coronary vessels on angiography characterizes the coronary slow flow phenomenon (CSFP) [1]. Although initially considered a benign condition, accumulating evidence suggests that CSFP is related to recurrent angina, myocardial ischemia, and unfavorable cardiovascular outcomes [2].

The exact biological basis of CSFP has not been fully elucidated and is hypothesized to be related to a complex interplay of microvascular dysfunction, endothelial impairment, inflammation, and early-stage atherosclerosis [3,4]. These mechanisms highlight the multifactorial nature of the condition and underscore the need for reliable biomarkers that reflect both metabolic and inflammatory processes.

Recently, growing interest has focused on novel metabolic and atherogenic indices as promising predictors of cardiovascular risk [5]. Among these, the triglyceride-glucose (TyG) index is increasingly considered a convenient and validated measure of insulin resistance (IR) and has been linked to a wide spectrum of cardiovascular conditions [6,7]. Accumulating evidence implies that an interaction exists between the TyG index and CSFP, highlighting a potential role of metabolic dysregulation in its pathogenesis [8,9].

Beyond metabolic dysfunction, nutritional and inflammatory status have gained increasing importance in cardiovascular risk stratification. Incorporating serum albumin and lymphocyte count, the Prognostic Nutritional Index (PNI) represents a combined measure reflecting both nutritional and immune function and has been implicated in poorer cardiovascular outcomes [10]. The C-reactive protein–albumin–lymphocyte (CALLY) index, Naples prognostic score (NPS), and Controlling Nutritional Status (CONUT) score, as composite immunonutritional indices, are also associated with adverse cardiovascular clinical endpoints, including increased mortality and poor prognosis, reflecting the combined impact of systemic inflammation, immune function, and nutritional balance [11,12,13]. Emerging data suggest that impaired nutritional status may also contribute to the pathogenesis of CSFP [14].

Despite these advances, most earlier studies have examined metabolic parameters and markers of nutritional status in isolation. However, considering the multifactorial nature of CSFP, the combined assessment of atherogenic, metabolic, and nutritional indices may allow a more integrated understanding of its pathophysiology. In particular, the TyG index, reflecting metabolic dysfunction and IR, and nutritional indices such as PNI, CONUT score, NPS, and CALLY index, reflecting inflammatory and nutritional status, may offer complementary information. Nevertheless, the integrated impact of these indices on CSFP, especially in non-elderly populations, remains insufficiently explored. Given that the early recognition of individuals at increased risk has the potential to improve clinical management, there is a need for integrative approaches that incorporate multiple pathophysiological pathways.

In this context, in individuals who underwent coronary angiography (CAG), we assessed the predictive value for the TyG index in combination with nutritional risk indices—including PNI, CONUT score, NPS, and the CALLY index—for CSFP in a non-elderly cohort.

## 2. Materials and Methods

### 2.1. Study Design and Population

A retrospective, single-center observational study was performed at Siirt Education and Research Hospital, a tertiary-level hospital in the southeastern region of Turkey. Consecutive patients who underwent CAG for suspected coronary artery disease (CAD), including stable angina, atypical chest pain, positive noninvasive ischemia testing, or suspected acute coronary syndrome (ACS), between January 2023 and December 2025 were screened for eligibility.

**Figure 1:**
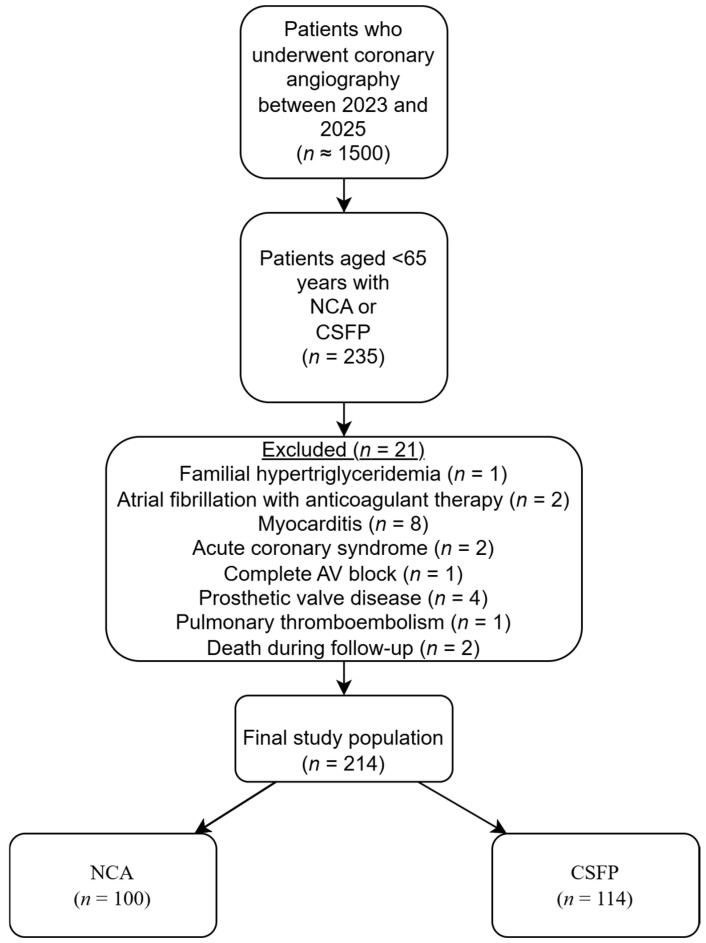
A flowchart of the selection of cases. NCA: normal coronary arteries; CSFP: coronary slow flow phenomenon.

A total of approximately 1500 patients were initially evaluated. Among them, 235 patients aged < 65 years with either angiographically normal coronary arteries (NCA) or CSFP, without obstructive CAD or significant valvular heart disease, were identified. A total of 21 patients were excluded according to predefined criteria, as a result of myocarditis (*n* = 8), prosthetic valve disease (*n* = 4), familial hypertriglyceridemia (*n* = 1), atrial fibrillation requiring anticoagulant therapy (*n* = 2), ACS (*n* = 2), complete atrioventricular block (*n* = 1), pulmonary thromboembolism (*n* = 1), and death during follow-up (*n* = 2). Overall, 214 patients were incorporated into this analysis, comprising 100 with NCA and 114 with CSFP (Figure 1).

This study was endorsed by the institutional ethics committee of Siirt University (approval number: 2026/01/04/7; date: 30 March 2026) and conformed to the Declaration of Helsinki. Owing to the study’s retrospective design, informed consent was not deemed necessary.

### 2.2. Clinical Definitions and Classification of Variables

Categorical variables and clinical definitions were established based on standard clinical criteria. Body mass index (BMI) reflects body weight (kg) adjusted for height squared (m^2^). Patients were classified as having diabetes mellitus (DM) if they had an established diagnosis or were receiving antidiabetic treatment, or if they had a fasting plasma glucose (FBG) level ≥ 126 mg/dL. Hypertension (HT) was considered present in patients with a known diagnosis, those who used antihypertensive agents, or with blood pressure values ≥ 140/90 mmHg on repeated assessments.

Smoking status was categorized as current or non-smoker based on self-reported information. Chronic obstructive pulmonary disease (COPD) was defined according to a prior clinical diagnosis documented in medical records or the use of bronchodilator therapy. Hypothyroidism was identified in patients with a documented diagnosis or those using thyroid hormone replacement therapy. Transthoracic echocardiographic examinations were performed according to current guideline recommendations, and left ventricular ejection fraction (LVEF) was calculated using the modified Simpson biplane method.

Baseline preprocedural pharmacotherapy profiles were retrospectively reviewed from medical records. The use of angiotensin-converting enzyme inhibitors/angiotensin receptor blockers (ACEI/ARB), beta-blockers, calcium channel blockers, oral antidiabetic agents, insulin therapy, and antiplatelet drugs before CAG was recorded and compared between the groups. Medications initiated after CAG or following the diagnosis of CSFP were intentionally excluded to minimize post-diagnostic treatment bias.

### 2.3. Evaluation of Coronary Flow Using TIMI Frame Count

The angiographic procedure was performed with the Artis Zee system, produced by Siemens Healthcare (Erlangen, Germany), via standard femoral or radial approaches, and image acquisition was performed at 15 frames per second (fps). The Gibson approach was used to derive the Thrombolysis in Myocardial Infarction (TIMI) frame count (TFC) values for the circumflex (CX), right coronary artery (RCA), and left anterior descending (LAD). The LAD value was corrected by applying a 1.7 adjustment factor. The initial frame was considered the moment when contrast fully opacified the coronary artery and contacted both vessel borders, while the final frame corresponded to the moment when the contrast reached predefined distal landmarks for each coronary artery. The mean TFC was obtained as the average of TFC values across three coronary arteries. Normal reference TFC values were accepted as 36 ± 2.5 frames for the LAD, 22.2 ± 4.1 frames for the CX artery, and 20.4 ± 3.1 frames for the RCA, as previously described by Gibson et al. [15]. CSFP was defined as delayed distal vessel opacification with TFC values exceeding 2 standard deviations above the established normal reference values in at least one major epicardial coronary artery in patients with angiographically NCA.

All angiographic measurements underwent independent analysis by two specialist cardiologists who were unaware of clinical and laboratory information. Patients were included in the CSFP and NCA groups only if both cardiologists were in agreement.

### 2.4. Laboratory Measurements

After a minimum fasting duration of 8 h, venous blood samples were obtained before CAG. Lipid profile components—including high-density lipoprotein cholesterol (HDL-C), triglycerides (TG), low-density lipoprotein cholesterol (LDL-C), and total cholesterol (TC)—and FBG, along with C-reactive protein (CRP), creatinine, and serum levels of albumin, were assessed using standard automated laboratory methods on a Beckman Coulter AU5800 analyzer (Beckman Coulter, Brea, CA, USA) in the hospital’s central laboratory. Enzymatic colorimetric assays were used for lipid parameters and glucose measurements, while CRP levels were quantified using immunoturbidimetric methods. Renal function was expressed as estimated glomerular filtration rate (eGFR) calculated with the Chronic Kidney Disease Epidemiology Collaboration (CKD-EPI) formula [16].

### 2.5. Atherogenic and Nutritional Indices

The TyG index was defined as ln [fasting TG (mg/dL) × FBG (mg/dL)/2]. Remnant cholesterol (RC) was obtained by deducting LDL-C and HDL-C from TC. The atherogenic coefficient (AC) was defined as the ratio of (TC − HDL-C) to HDL-C. The atherogenic index of plasma (AIP) was expressed as log (TG/HDL-C). The Castelli Risk Index I (CRI-I) and II (CRI-II) were expressed as the ratios of TC to HDL-C and LDL-C to HDL-C, respectively.

Inflammatory and nutritional indices were calculated as follows.

The neutrophil-to-lymphocyte ratio (NLR) was calculated as the absolute neutrophil count divided by the lymphocyte count, whereas the lymphocyte-to-monocyte ratio (LMR) was defined as the lymphocyte count divided by the monocyte count. The PNI was derived using the formula: 10 × serum albumin (g/dL) + 0.005 × lymphocyte count (/mm^3^). The CONUT score was determined based on serum albumin, TC, and lymphocyte count. Scoring for each parameter was performed according to predefined thresholds: serum albumin ≥ 3.5 g/dL = 0, 3.0–3.49 = 2, 2.5–2.99 = 4 and <2.5 = 6 points; TC ≥ 180 mg/dL = 0, 140–179 = 1, 100–139 = 2, and <100 = 3 points; lymphocyte count ≥ 1600/mm^3^ = 0, 1200–1599 = 1, 800–1199 = 2, and <800 = 3 points. The NPS was built on four parameters: NLR, serum albumin, TC, and LMR. Each parameter was assigned 1 point according to predefined cut-off values: NLR > 2.96 = 1 point, ≤2.96 = 0 points; serum albumin < 4.0 g/dL = 1 point, ≥4.0 g/dL = 0 points; TC < 180 mg/dL = 1 point, ≥180 mg/dL = 0 points; and LMR < 4.44 = 1 point, ≥4.44 = 0 points. The total NPS varied from 0 to 4, with increasing scores representing greater impairment in nutritional and inflammatory status. The CALLY index was derived as a composite measure integrating inflammatory and nutritional parameters, calculated as (albumin × lymphocyte count)/CRP. Higher values reflect a more favorable nutritional and inflammatory profile. All indices were calculated using previously validated methods described in the literature [17,18]. A schematic overview summarizing the formulas and scoring systems of the evaluated metabolic and immunonutritional indices is provided in Supplementary Figure S1.

### 2.6. Statistical Analysis

All statistical analyses were performed with SPSS software (version 26.0; IBM Corp., Armonk, NY, USA). Continuous data are expressed as mean ± standard deviation (SD), while categorical variables are summarized as counts and percentages. Data distribution was evaluated for normality using the Kolmogorov–Smirnov test along with visual histogram assessment. Since the data were normally distributed, group comparisons were utilized using the independent samples *t*-test, while categorical variables were evaluated with Pearson’s chi-square test.

Univariate logistic regression was employed to evaluate factors related to CSFP. Covariates with *p* < 0.10 were incorporated into the multivariable logistic regression model. To improve comparability and reduce the effect of scale differences, selected continuous variables (TyG index and PNI) were standardized using z-score transformation prior to multivariable analysis. Results are reported as odds ratios (ORs) with corresponding 95% confidence intervals (CIs). The Hosmer–Lemeshow goodness-of-fit test was applied to examine model calibration. The explanatory capacity of the model was investigated using Cox & Snell and Nagelkerke R^2^ values.

The discriminative ability of the TyG index, PNI, and the multivariable model was assessed with receiver operating characteristic (ROC) curve analysis. The area under the curve (AUC) values were expressed with 95% confidence intervals. Using the Youden index, optimal cut-off points were selected, with sensitivity and specificity reported accordingly.

Results with a two-sided *p* value < 0.05 were interpreted as statistically relevant.

## 3. Results

### 3.1. Baseline Clinical and Demographic Profile of the Study Population

The final study cohort comprised 214 patients, including 100 with NCA and 114 with CSFP. The groups were comparable in terms of age (50.98 ± 8.16 vs. 53.03 ± 8.23 years, *p* = 0.070) or BMI (27.45 ± 4.12 vs. 28.15 ± 4.02 kg/m^2^, *p* = 0.209). LVEF values were within normal limits in both groups and did not differ significantly between the groups (*p* = 0.322). Male sex was significantly more common in the CSFP group compared with the NCA group (73.7% vs. 43.0%, *p* < 0.001). Similarly, smoking was encountered more often in the CSFP group (33.3% vs. 19.0%, *p* = 0.018). HT, DM, COPD, and hypothyroidism were comparable between the groups. No statistically significant differences were observed regarding preprocedural use of ACEI/ARB, beta-blockers, calcium channel blockers, oral antidiabetic agents, insulin therapy, or antiplatelet drugs between patients with CSFP and normal coronary arteries (Table 1).

### 3.2. Laboratory and Angiographic Findings

Patients with CSFP had significantly higher TG levels than those with NCA (167.59 ± 71.92 vs. 128.79 ± 58.97 mg/dL, *p* < 0.001). Albumin levels were notably lower in the CSFP group (4.24 ± 0.33 vs. 4.38 ± 0.26 g/dL, *p* = 0.001). In addition, creatinine and CRP levels were markedly higher in the CSFP group (both *p* < 0.05), while eGFR values were significantly lower (94.73 ± 9.61 vs. 98.12 ± 10.05 mL/min/1.73 m^2^, *p* = 0.013). Comparisons between the groups revealed no significant differences in TC, HDL-C, LDL-C, neutrophil count, or lymphocyte count (all *p* > 0.05). All TIMI frame count indices—Corrected LAD, LAD, CX, RCA, and mean TFC—were greater in the CSFP group relative to the NCA group (all *p* < 0.001) (Table 2).

### 3.3. Atherogenic, Metabolic, and Nutritional Indices

The TyG index and AIP showed higher values in the CSFP group than in the NCA group (both *p* < 0.001). In contrast, the PNI and CALLY scores were significantly reduced in the CSFP group (*p* = 0.002 and *p* = 0.024, respectively). The NLR was further increased in patients with CSFP (*p* = 0.045). Similar values were observed between the groups for RC, CRI-I, CRI-II, AC, LMR, NPS, and CONUT score (all *p* > 0.05) (Table 3).

### 3.4. Analysis of Independent Predictors of CSFP

**Figure 2:**
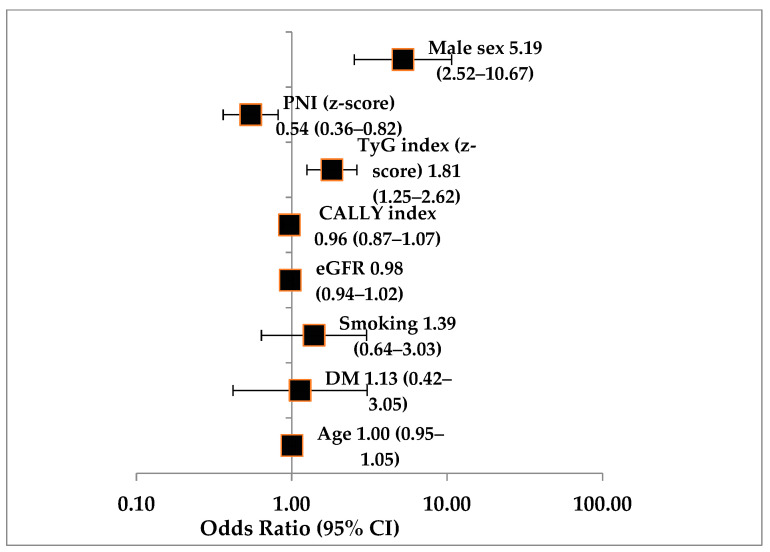
Forest plot of multivariable logistic regression analysis identifying predictors of coronary slow flow.

In univariate logistic regression analysis, smoking (OR = 2.132, 95% CI: 1.131–4.016, *p* = 0.019), eGFR (OR = 0.965, 95% CI: 0.938–0.993, *p* = 0.014), CALLY index (OR = 0.907, 95% CI: 0.831–0.990, *p* = 0.028), TyG index (z-score) (OR = 1.894, 95% CI: 1.397–2.568, *p* < 0.001), PNI (z-score) (OR = 0.646, 95% CI: 0.481–0.868, *p* = 0.004), and male sex (OR = 3.712, 95% CI: 2.089–6.595, *p* < 0.001) were significantly associated with CSFP (Table 4). All variables meeting the *p* < 0.10 threshold in univariate analysis, such as age, DM, smoking, eGFR, CALLY index, TyG index (z-score), PNI (z-score), and male sex, were incorporated into the multivariable logistic regression model (Figure 2).

In multivariable analysis, the TyG index (z-score) (OR = 1.811, 95% CI: 1.251–2.622, *p* = 0.002), PNI (z-score) (OR = 0.544, 95% CI: 0.362–0.817, *p* = 0.003), and male sex (OR = 5.187, 95% CI: 2.520–10.674, *p* < 0.001) persisted as independent predictors of CSFP (Table 4). The multivariable model achieved good calibration (Hosmer–Lemeshow *p* = 0.474), moderate explanatory power (Nagelkerke R^2^ = 0.325), and an overall classification accuracy of 69.6%.

### 3.5. Diagnostic Performance of Predictive Models for CSFP

**Figure 3:**
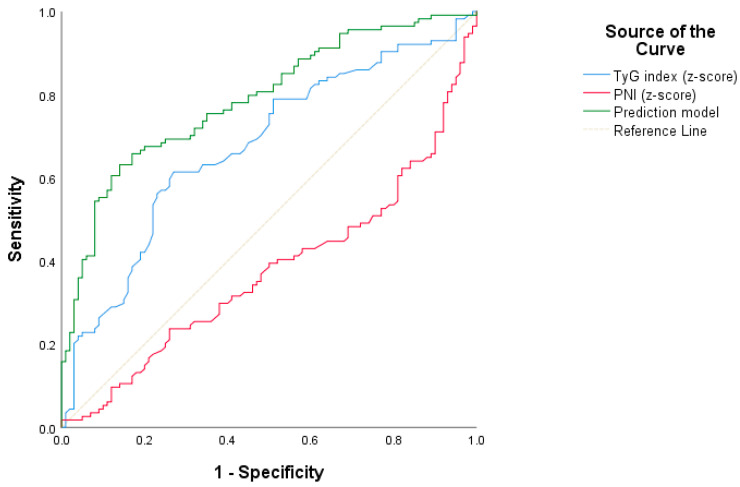
Receiver operating characteristic (ROC) curves of TyG index, PNI, and the multivariable prediction model for identifying coronary slow flow.

The discriminative performance of the TyG index, PNI, and the multivariable logistic regression model in predicting CSFP was assessed using ROC curve analysis (Figure 3). The TyG index provided moderate discriminative capacity (AUC = 0.675, 95% CI: 0.603–0.747, *p* < 0.001), whereas PNI showed limited discriminative ability (AUC = 0.376, 95% CI: 0.301–0.450, *p* = 0.002).

The multivariable model showed good predictive performance with an AUC of 0.789 (95% CI: 0.729–0.849, *p* < 0.001). The optimal cut-off value for the TyG index was 8.68, yielding a sensitivity of 74.6% and a specificity of 50.0% (Table 5).

## 4. Discussion

The current analysis provides novel and clinically meaningful evidence that metabolic and immunonutritional pathways jointly contribute to the development of CSFP. Importantly, this study appears to be the first to demonstrate the independent and complementary predictive performance of both the TyG index and PNI in patients with CSFP. Additionally, male sex emerged as a strong independent predictor, further supporting the contribution of sex-related differences in cardiovascular risk profile and endothelial function to the pathogenesis of CSFP.

Although the coronary slow flow phenomenon is frequently underestimated in daily clinical practice and often considered a relatively benign angiographic finding, it may lead to recurrent chest pain episodes resulting in repeated emergency department admissions, impaired quality of life, and occasionally serious rhythm and conduction disturbances [19]. Accordingly, CSFP should be recognized as a clinically relevant entity requiring further investigation regarding its underlying pathophysiological mechanisms and therapeutic management.

Accumulating evidence suggests that the coronary slow flow phenomenon is not merely a localized coronary microvascular disorder but rather a systemic condition involving IR and endothelial impairment, accompanied by ongoing low-grade inflammation [20]. In this context, the TyG index has gained increasing attention as a widely accepted reflector of IR and metabolic burden [21]. Earlier studies have revealed a meaningful correlation between the TyG index and coronary microvascular dysfunction [22,23]. Consistent with these findings, our results reinforce the central role of metabolic dysregulation in CSFP.

Beyond metabolic factors, nutritional and inflammatory status have gained importance as key determinants of cardiovascular risk [24]. The PNI is a well-established marker reflecting the interplay between immune and nutritional status. Prior research has suggested that reduced PNI levels are related to increased inflammatory burden, endothelial dysfunction, and adverse cardiovascular outcomes [25]. Mechanistically, reduced albumin levels may promote systemic inflammation [26], while lymphocytopenia reflects impaired immune regulation [27], both of which contribute to vascular dysfunction.

In the context of CSFP, recent evidence indicates that lower PNI levels independently predict impaired coronary flow, emphasizing the involvement of systemic inflammation and nutritional alterations in microvascular dysfunction [14]. In our study, the albumin-containing PNI retained its status as an independent predictor of CSFP. Considering that serum albumin levels are generally preserved in individuals without advanced comorbid conditions [26], lower PNI values among this cohort may represent subtle inflammatory and nutritional alterations rather than overt malnutrition. This finding suggests that even in relatively lower-risk populations, early systemic disturbances may contribute to coronary microvascular dysfunction.

Moreover, compared to other composite indices such as CONUT score, NPS, and CALLY index, PNI may offer a more direct and parsimonious assessment of the immune–nutritional axis with less redundancy among its components. This may explain why PNI retained its independent predictive value in our multivariable model, whereas other indices did not, highlighting its potential utility as a practical and clinically meaningful marker in CSFP.

Existing studies have highlighted how well the TyG index predicts CSFP, primarily focusing on its association with metabolic and hematological parameters. Yüksel et al. observed that the TyG index, along with HDL-C and hemoglobin levels, was an independent predictor of CSFP, highlighting the contribution of metabolic and hematologic factors to impaired coronary flow [28]. Similarly, Kaplangoray et al. found that male sex, TyG index, BMI, and NLR were independently associated with CSFP, emphasizing the contribution of metabolic burden, inflammatory processes, and sex-specific differences [29].

In line with these findings, our results also confirm the independent predictive roles of the TyG index and male sex with CSFP. However, our study extends the existing literature by providing novel evidence that the PNI, a marker increasingly recognized in recent cardiovascular research, is an independent predictor of CSFP when evaluated together with the TyG index. Recent studies further support the integrative evaluation of metabolic and inflammatory–nutritional indices in cardiovascular disease. A composite index combining the TyG index and PNI, known as the insulin resistance–nutritional index (IRNI), has been found to enhance clinical outcomes related to mortality in diabetic heart failure patients compared to individual markers alone [30]. Similarly, Zhu et al. demonstrated that a model incorporating TyG index and PNI provided enhanced predictive performance for acute kidney injury occurring after coronary intervention due to contrast exposure, highlighting the synergistic interaction between metabolic dysfunction and nutritional–inflammatory status [31]. Importantly, while these integrative approaches have been applied in advanced cardiovascular conditions and procedure-related complications, their role in coronary microvascular dysfunction, particularly in CSFP, has not been previously investigated. Moreover, the combined model incorporating TyG index, PNI, and male sex showed enhanced predictive performance, suggesting that a composite evaluation integrating metabolic, inflammatory–nutritional, and demographic factors may aid in identifying patients with an increased likelihood of CSFP through a more comprehensive and clinically relevant approach.

In addition to these findings, several noteworthy observations from our analysis merit further consideration. In our analysis, although the AIP was significantly higher in patients with CSFP, it was omitted from the multivariable analysis owing to potential collinearity with the TyG index. Given that both indices are derived from TG levels and reflect overlapping metabolic pathways, inclusion of both variables in the same model may have introduced multicollinearity and compromised the stability of the model estimates. Therefore, the TyG index, which has been more consistently associated with IR and microvascular dysfunction, was preferred in the final model.

A key advantage of this analysis is the inclusion of patients with angiographically NCA and no visible atherosclerotic plaque. This strict selection may have minimized the confounding effects of subclinical atherosclerosis and allowed a more accurate evaluation of functional microvascular impairment. In this context, the lack of independent predictive value of traditional atherogenic indices further supports the notion that CSFP is primarily driven by functional rather than structural vascular abnormalities.

Interestingly, conventional cardiovascular risk factors, including age, DM, and smoking, did not emerge as independent predictors in our analysis. This observation may reflect the relatively homogeneous and lower-risk study population, as well as the exclusion of patients with overt CAD. It also suggests that, in the setting of CSFP, functional and metabolic disturbances may play a more prominent role than conventional risk factors, particularly in earlier or subclinical stages of cardiovascular disease.

From a diagnostic perspective, the TyG index showed moderate discriminative ability, while PNI alone demonstrated limited performance, likely reflecting its inverse relationship with CSFP. In contrast, the combined multivariable model incorporating TyG index, PNI, and male sex demonstrated good predictive performance. This finding highlights the advantage of integrating multiple pathophysiological domains. Given the multifactorial nature of CSFP, a single biomarker may not adequately reflect disease complexity, whereas a combined approach that captures metabolic, inflammatory–nutritional, and demographic factors may optimize risk stratification and inform clinical decisions. Notably, the inclusion of a non-elderly population represents an additional strength of our study, as it allows the evaluation of CSFP at an earlier stage of cardiovascular risk, minimizing the confounding effects of advanced age and accumulated comorbidities.

### 4.1. Limitations

A number of limitations should be taken into account. Being retrospective in design, this single-center study may constrain the wider applicability of the observed results. Another important point is that causal relationships cannot be established owing to the study’s observational design. Moreover, although multiple metabolic and nutritional indices were evaluated, other inflammatory biomarkers were not included. Additionally, alternative techniques for evaluating coronary flow and microvascular dysfunction, such as coronary flow reserve (CFR), index of microcirculatory resistance (IMR), Doppler-derived intracoronary flow measurements, and PET-based perfusion imaging, were not available in this retrospective study setting; therefore, coronary microvascular function was inferred rather than directly assessed. Furthermore, although all patients had angiographically normal coronary arteries without visible atherosclerotic plaque, the absence of imaging methods, specifically optical coherence tomography (OCT) and intravascular ultrasound (IVUS), may have limited the detection of subclinical atherosclerosis. Finally, the corroboration of these findings requires further validation in broader prospective, multicenter settings.

### 4.2. Future Directions

Prospective investigations with expanded cohorts are required to corroborate these empirical findings and investigate whether combined metabolic–nutritional indices can improve clinical outcomes. In addition, further mechanistic investigations are required to further elucidate the interaction between IR, inflammation, and microvascular dysfunction in CSFP. The development of integrated risk models incorporating both metabolic and nutritional parameters may provide new opportunities for early diagnosis and targeted therapeutic strategies.

Furthermore, future research may investigate whether dynamic changes in these indices over time are associated with disease progression or therapeutic response. In addition, integrating multimarker approaches with advanced imaging or functional assessments of the coronary microcirculation may further enhance risk stratification and deepen our understanding of CSFP pathophysiology.

## 5. Conclusions

Growing evidence suggests that the coronary slow flow phenomenon represents a complex clinical entity extending beyond simple microvascular impairment. In this context, our findings indicate that metabolic dysfunction, immunonutritional alterations, and sex-related differences jointly contribute to its pathophysiology in a non-elderly population.

The combined assessment of the TyG index, PNI, and male sex provides a more comprehensive and clinically meaningful approach for identifying patients at risk. Collectively, these results indicate the potential relevance of integrating metabolic and immunonutritional markers with clinical characteristics for improved risk stratification and may contribute to a better understanding of coronary microvascular dysfunction.

## Figures and Tables

**Table 1:** Baseline demographic and clinical characteristics of the study population.

Variables	NCA (*n* = 100)	CSFP (*n* = 114)	*p*-Value
Age (years)	50.98 ± 8.16	53.03 ± 8.23	0.070
BMI (kg/m^2^)	27.45 ± 4.12	28.15 ± 4.02	0.209
EF (%)	60.61 ± 4.60	59.94 ± 5.21	0.322
Male sex, *n* (%)	43 (43.0%)	84 (73.7%)	<0.001
HT, *n* (%)	35 (35.0%)	50 (43.9%)	0.186
DM, *n* (%)	13 (13.0%)	26 (22.8%)	0.064
Smoking, *n* (%)	19 (19.0%)	38 (33.3%)	0.018
COPD, *n* (%)	9 (9.0%)	15 (13.2%)	0.336
Hypothyroidism, *n* (%)	8 (8.0%)	6 (5.3%)	0.419
Baseline pharmacotherapy			
ACEI/ARB use, *n* (%)	29 (29.0%)	39 (34.2%)	0.414
Beta-blocker use, *n* (%)	11 (11.0%)	21 (18.4%)	0.129
CCB use, *n* (%)	7 (7.0%)	11 (9.6%)	0.486
Oral antidiabetic use, *n* (%)	13 (13.0%)	26 (22.8%)	0.064
Insulin use, *n* (%)	1 (1.0%)	5 (4.4%)	0.134
Antiplatelet use, *n* (%)	28 (28.0%)	33 (28.9%)	0.878

Data are presented as mean ± standard deviation for continuous variables and number (percentage) for categorical variables. Continuous variables were compared using the independent samples *t*-test, while categorical variables were analyzed using the chi-square test. Abbreviations: NCA—normal coronary arteries; CSFP—coronary slow flow phenomenon; BMI—body mass index; EF—ejection fraction; HT—hypertension; DM—diabetes mellitus; COPD—chronic obstructive pulmonary disease; ACEI/ARB—angiotensin-converting enzyme inhibitor/angiotensin receptor blocker; CCB—calcium channel blocker.

**Table 2:** Laboratory parameters and coronary angiographic characteristics of patients with NCA and CSFP.

Variables	NCA (*n* = 100)	CSFP (*n* = 114)	*p*-Value
Glucose (mg/dL)	105.60 ± 33.26	114.07 ± 38.89	0.087
Triglycerides (mg/dL)	128.79 ± 58.97	167.59 ± 71.92	<0.001
Total cholesterol (mg/dL)	184.68 ± 35.00	187.59 ± 36.94	0.557
HDL-C (mg/dL)	52.14 ± 12.38	49.77 ± 12.72	0.170
LDL-C (mg/dL)	106.78 ± 28.87	108.69 ± 27.99	0.623
Albumin (g/dL)	4.38 ± 0.26	4.24 ± 0.33	0.001
Creatinine (mg/dL)	0.78 ± 0.13	0.86 ± 0.12	<0.001
eGFR (mL/min/1.73 m^2^)	98.12 ± 10.05	94.73 ± 9.61	0.013
CRP (mg/L)	2.49 ± 1.09	2.85 ± 1.19	0.023
Neutrophil count (×10^3^/µL)	4.38 ± 1.29	4.63 ± 1.58	0.204
Lymphocyte count (×10^3^/µL)	2.63 ± 0.78	2.47 ± 0.84	0.157
Corrected LAD TFC	22.04 ± 5.40	35.86 ± 6.59	<0.001
LAD TFC	37.47 ± 9.19	60.96 ± 11.20	<0.001
LCX TFC	22.50 ± 6.33	36.72 ± 9.02	<0.001
RCA TFC	20.61 ± 6.32	34.36 ± 8.02	<0.001
Mean TFC	21.72 ± 4.29	35.65 ± 5.15	<0.001

Data are presented as mean ± standard deviation. Continuous variables were compared between groups using the independent samples *t*-test. Abbreviations: NCA—normal coronary arteries; CSFP—coronary slow flow phenomenon; HDL-C—high-density lipoprotein cholesterol; LDL-C—low-density lipoprotein cholesterol; eGFR—estimated glomerular filtration rate; CRP—C-reactive protein; LAD—left anterior descending artery; LCX—left circumflex artery; RCA—right coronary artery; TFC—TIMI frame count.

**Table 3:** Comparison of atherogenic, metabolic, and nutritional risk indices between patients with NCA and CSFP.

Variables	NCA (*n* = 100)	CSFP (*n* = 114)	*p*-Value
TyG index	8.71 ± 0.49	9.02 ± 0.55	<0.001
AIP	0.37 ± 0.21	0.50 ± 0.23	<0.001
RC (mg/dL)	25.57 ± 16.10	29.13 ± 20.38	0.156
CRI-I	3.69 ± 0.95	3.93 ± 1.01	0.076
CRI-II	2.16 ± 0.79	2.29 ± 0.73	0.222
AC	2.69 ± 0.95	2.93 ± 1.01	0.076
NLR	1.79 ± 0.74	2.04 ± 1.06	0.045
LMR	6.11 ± 2.92	5.61 ± 1.93	0.149
Naples score	0.91 ± 0.85	1.10 ± 0.98	0.138
CONUT score	0.56 ± 0.57	0.55 ± 0.68	0.932
PNI	56.92 ± 4.70	54.72 ± 5.77	0.002
CALLY index	5.63 ± 3.46	4.58 ± 3.25	0.024

Data are presented as mean ± standard deviation. Continuous variables were compared between groups using the independent samples *t*-test. Abbreviations: NCA—normal coronary arteries; CSFP—coronary slow flow phenomenon; TyG—triglyceride-glucose index; AIP—atherogenic index of plasma; RC—remnant cholesterol; CRI-I—Castelli Risk Index I; CRI-II—Castelli Risk Index II; AC—atherogenic coefficient; NLR—neutrophil-to-lymphocyte ratio; LMR—lymphocyte-to-monocyte ratio; CONUT—Controlling Nutritional Status; PNI—Prognostic Nutritional Index; CALLY—C-reactive protein–albumin–lymphocyte.

**Table 4:** Univariate and multivariable logistic regression analyses for predictors of CSFP.

Variable	Univariate OR (95% CI)	*p*-Value	Multivariate OR (95% CI)	*p*-Value
Age	1.031 (0.997–1.066)	0.071	1.002 (0.953–1.053)	0.932
DM	1.977 (0.954–4.098)	0.067	1.129 (0.419–3.046)	0.810
Smoking	2.132 (1.131–4.016)	0.019	1.389 (0.637–3.030)	0.409
eGFR (mL/min/1.73 m^2^)	0.965 (0.938–0.993)	0.014	0.977 (0.937–1.018)	0.269
CALLY index	0.907 (0.831–0.990)	0.028	0.963 (0.868–1.068)	0.473
TyG index (z-score)	1.894 (1.397–2.568)	<0.001	1.811 (1.251–2.622)	0.002
PNI (z-score)	0.646 (0.481–0.868)	0.004	0.544 (0.362–0.817)	0.003
Male sex	3.712 (2.089–6.595)	<0.001	5.187 (2.520–10.674)	<0.001

Variables with *p* < 0.10 in univariate analysis were entered into the multivariable logistic regression model. Odds ratios (ORs) and 95% confidence intervals (CIs) were calculated for each variable. Abbreviations: OR—odds ratio; CI—confidence interval; DM—diabetes mellitus; eGFR—estimated glomerular filtration rate; TyG—triglyceride-glucose index; PNI—Prognostic Nutritional Index; CALLY—C-reactive protein–albumin–lymphocyte.

**Table 5:** Receiver operating characteristic (ROC) curve analysis for predicting CSFP.

Variables	AUC	95% CI	*p*-Value	Cut-Off	Sensitivity (%)	Specificity (%)
TyG index	0.675	0.603–0.747	<0.001	8.68	74.6	50
PNI	0.376	0.301–0.450	0.002	54	53.5	20
Predicted probability (multivariable model)	0.789	0.729–0.849	<0.001	0.5	71.1	68

Abbreviations: AUC—area under the curve; CI—confidence interval; ROC—receiver operating characteristic; TyG—triglyceride-glucose index; PNI—Prognostic Nutritional Index; CSFP—coronary slow flow phenomenon.

## Data Availability

The datasets generated and analyzed during the current study are available from the corresponding author upon reasonable request.

## Supplementary Materials

The following supporting information can be downloaded at https://www.mdpi.com/article/10.3390/jcm15114004/s1, Figure S1: Overview of Atherogenic, Metabolic, Inflammatory, and Immunonutritional Indices Used in the Study.
