# Supplementary figures and images for: Combined Assessment of Immunonutritional Indices and the Triglyceride-Glucose Index in Coronary Slow Flow Phenomenon in a Non-Elderly Population
Source: J Clin Med. 2026 May 22;15(11):4004. doi: 10.3390/jcm15114004 (PMC13258067; doi:10.3390/jcm15114004)

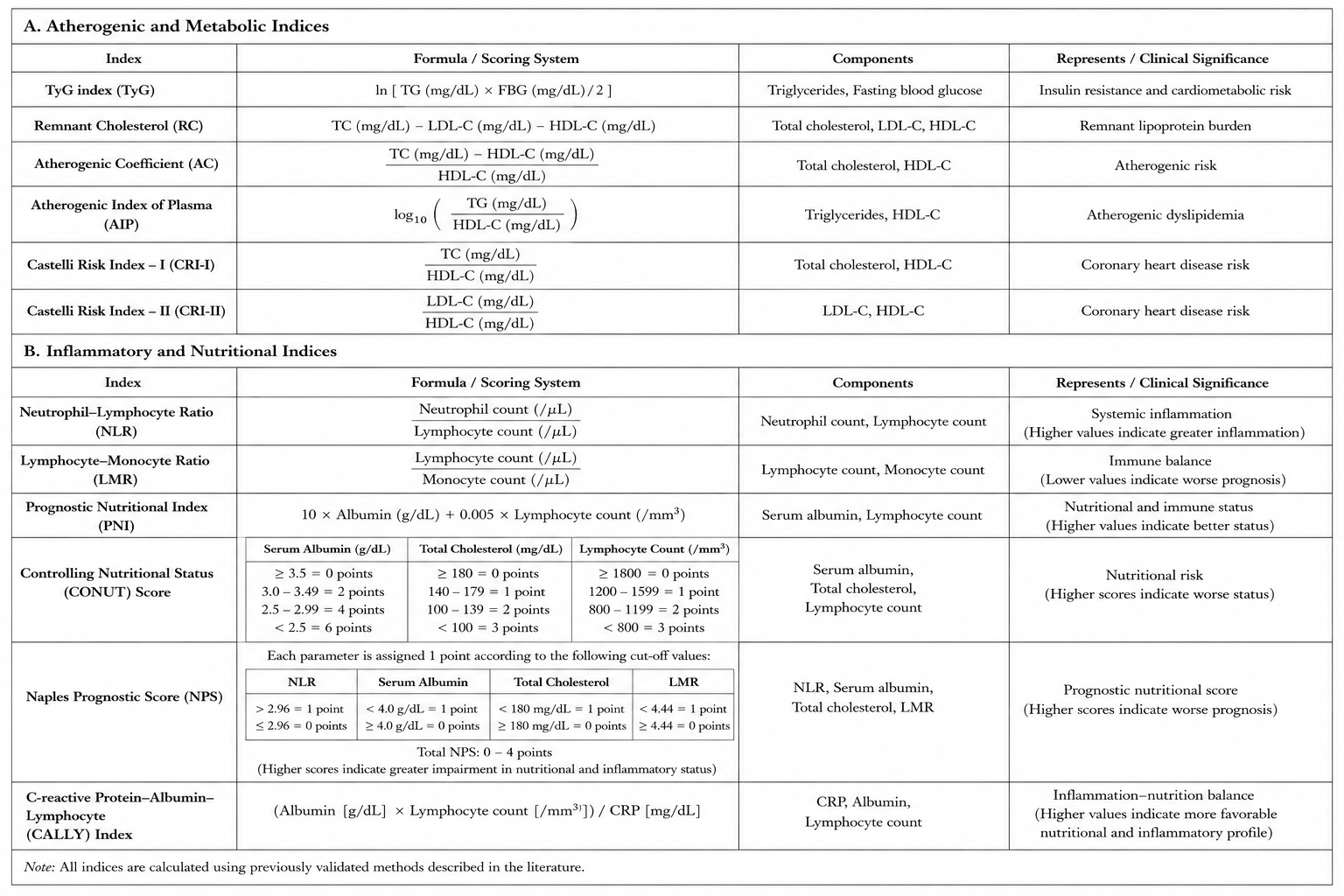

Supplement: Supplementary file 1 [file jcm-15-04004-s001.zip › jcm-4312518-supplementary.png]
